# Supplementary material for: Conjugative Plasmid pPPUT-Tik1-1 from a Permafrost Pseudomonas putida Strain and Its Present-Day Counterparts Inhabiting Environments and Clinics
Source: Int J Mol Sci. 2023 Aug 31;24(17):13518. doi: 10.3390/ijms241713518 (PMC10488154; doi:10.3390/ijms241713518)
Supplement: Supplementary file 1 [file ijms-24-13518-s001.zip › Table S1.pdf]

**Table S1.** Characteristics of the *Pseudomonas* plasmids related to pTik1-1

| Strain | Plasmid | Size (bp) | Source | *repA2* | *mobEDCBA* | *traYWUTRQPONM* | *repA1* | Accessory region | | Accession Number |
|---|---|---|---|---|---|---|---|---|---|---|
| | | | | | | | | resistance genes | other | |
| *P. putida* Tik1 | pPPUT-Tik1-1 | 153663 | permafrost | 100% | 100% | 100% | 100% | *mer*[1], *strAB* | - | OQ920555 |
| *P. putida* 15420352 | p420352str | 153678 | urine, China | 100% | 99,9% | 100% | 99,9% | *mer*[1], *strAB* | - | MT74087 |
| *P.fulva* ZDHY414 | Unnamed | 150273 | clinic, China | 100% | 100% | 100% | 100% | *mer*[1], *strAB* | - | CP064947 |
| *P. putida* 12969 | p12969-2 | 109708 | clinic, China | 88,8% | 86,8% | 85,8% | 86,4% | *mer*[1], *sul1, aadA* | - | KY270855 |
| *P. putida* 1290 | pPp1290 | 114265 | pear pyllosphere | 100% | 99,9% | 99,9% | 99,9% | - | aromatic compounds degradation | CP039372 |
| *P. putida* DOT-T1E | pGRT1 | 133451 | unknown | 95,3% | 97,1% | 88,0% | 96,2% | UV-resistance | tolerance to toluene (ttgGHI) | HM626202 |
| *P. juntendi* 18091276 | pCN1276 | 112529 | urine, China | 92,2% | 87,2% | 86,0% | 91,9% | *mer*[1] | - | CP091312 |
| *P. monteilii* B5 | pSH5-1 | 130536 | soil | 92,1% | 87,2% | 86,0% | 91,9% | Δ*mer*[2] | - | CP022562 |
| *Pseudomonas* sp.BYT-5 | pBYT5-2 | 136400 | soil | 95,4% | 96,2% | 98,1% | 98,1% | - | Molybdate absorbtion | CP097489 |
| *P. syringae* pv. actinidiae S7-1M | pS7-1M | 68717 | kiwifruit orchard, New Zealand | 84,7% | 79,6% | 81,8% | 80,5 | - | - | MK621014 |
| *P. putida* | pPp-DIM | 69823 | water, Spain | 92,3% | - | - | 91,1% | GNAT, *sul1- aadB*, *tetA(R)*, *bla*DIM1 | - | LR822050 |
| *P.monteilii* | unnamed | 60588 | sputum, USA | 92,3% | - | - | 91,2% | Δ*mer*[2] | - | CP014061 |
| *P.monteilii* STW0522-72 | pSTW0522-72-2 | 59057 | hospital sewage, Japan | 91,9% | - | - | 91,1% | - | - | AP022475 |

[1] *mer*-operon, [2] only *merRTP* is present
