# Supplementary material for: Conjugative Plasmid pPPUT-Tik1-1 from a Permafrost Pseudomonas putida Strain and Its Present-Day Counterparts Inhabiting Environments and Clinics
Source: Int J Mol Sci. 2023 Aug 31;24(17):13518. doi: 10.3390/ijms241713518 (PMC10488154; doi:10.3390/ijms241713518)
Supplement: Supplementary file 1 [file ijms-24-13518-s001.zip › Table S2.pdf]

Table S2. Identification of the replicon type of pPPUT-Tik1-1

Inc group	Reference Plasmid	AC	*repA1*		*repA2*	
			**Identity**	**cover**	**Identity**	**Cover**
IncP-1	R751	AP002527.1	absent	-	absent	-
IncP-2	Rms139*	LC653116.1	37.2%	46. 7%	absent	-
	pOZ176*	NC_022344.1	38.7%	12%	31.6%	49%
IncP-3	pRA1	FJ705807.1	absent	-	85.3%	3%
IncP-3-like	pNK546a	MN433457.1	39%	12%	39%	11%
	pQBR103	NC_009444.1	52%	5%	absent	-
IncP-4 (Q)	RSF1010	NC_001740.1	absent	-	absent	-
IncP-6	Rms149	AJ877225.1	88%	2%	32%	11%
	p10265-KPC	KU578314.1	36%	27%	absent	-
IncU	pD5170990	KX169264.1	37%	20%	41%	35%
IncP-7	pND6-1	AY208917.1	41%	6%	45%	24%
	pCAR1	NC_004444.1	75,0%	11%	35%	51%
IncP-9	pWWO	AJ344068.1	48%	6%	absent	-
	pDTG1	NC_004999.1	41%	6%	42%	8%
IncP-10	R91-5	X54695.1	absent	-	absent	-
IncP-11**	pOXA-198	MG958650.1	**72%**	**88%**	37%	10%
	pRSB101	AJ698325.1	**72%**	**92%**	absent	-
	pPAO33	CP089239.1	**72%**	**96%**	43%	17%
pPT23A	pPS0081	KY362368.1	**66.7%**	**84%**	50%	32%
	pPS7B44	KY362373.1	**66.7%**	**90%**	38.9%	40%
IncpSTY-1	pSTY	NC_022739.1	48%	6%	40%	12%
IncpSTY-2	p716811-VIM	MN310372.1	absent	-	30.4%	14%
IncpSTY-3	pHN39-SIM	KU254577.1	34.8	16%	55.6	5%

*plasmid Rms139 contains a single *rep* gene (*repP-2A*), while plasmid pOZ176 contains two *rep* genes; **proposed by Bonnin et al. [11].
