# Supplementary material for: Conjugative Plasmid pPPUT-Tik1-1 from a Permafrost Pseudomonas putida Strain and Its Present-Day Counterparts Inhabiting Environments and Clinics
Source: Int J Mol Sci. 2023 Aug 31;24(17):13518. doi: 10.3390/ijms241713518 (PMC10488154; doi:10.3390/ijms241713518)
Supplement: Supplementary file 1 [file ijms-24-13518-s001.zip › Table S3.pdf]

Table S3. List of pPPUT-Tik1-1 homologs found in whole shortgun contigs sequences

**Accession number**	**Species**	**Strain**	**Contig**	**Size**	**Source**	**Accessory modules**
CAIGJX010000016.1<br>CAIGJX010000128.1	*P. putida*	Isolate 8	NODE16<br>NODE128	89894<br>10532	*n*d	solvent (toluene) tolerance
LSUZ01000124.1	*P. putida*	INSali382<br>PpAli382	21	87257	vegetable	
JAJSQY010000040.1<br>JAJSQY010000041.1	*P. putida*	NMI5241_13	NODE40<br>NODE41	57001<br>53270	Homo sapience	
JADUCG010000055.1<br>JADUCG010000036.1	*P. putida*	PSB00025	c0055<br>c0036	39438<br>59934	Homo sapience	Tn*5563a*
LVHH01000062.1<br>LVHH01000087.1	*P. putida*	KB9	scaffold57<br>scaffold8.1	32054<br>304965	apoplast from Arabidopsis thaliana	Tn*5563a*
JAEHTH010000069.1<br>JAEHTH010000022.1	*P. putida*	PSB00039	c0069<br>c0022	22362<br>87727	Homo sapience	ΔTn*501**<br>Tn*5563a*
JAEHTG010000065.1<br>JAEHTG010000035.1	*P. putida*	PSB00040	c0065<br>c0035	22286<br>65334	Homo sapience	ΔTn*501**
JADUCP010000013.1	*P. putida*	PSB00012	c0013	146218	Homo sapience	ΔTn*501**<br>Tn*5563a*<br>*chrAB*
RJAI01000012.1<br>RJAI01000007.1<br>RJAI01000117.1	*P. putida*	12917	contig_12<br>contig_7<br>contig_11	52850<br>21999<br>11087	Homo sapience	Tn*5041*
22JAJSQT010000015.1	*P. alloputida*	NMI6396_12	NODE_15	145154	Homo sapience	-
JAJSRL010000007.1	*P. alloputida*	NMI3760_14	NODE_7	135534	Homo sapience	Tn*5563a*

JAJSRH010000059.1<br>JAJSRH010000025.1	*P. alloputida*	NMI733_14	NODE_59<br>NODE_25	22202<br>98021	Homo sapience	Tn*5563a*
JAJSQJ010000044.1<br>JAJSQJ010000033.1	*P. monteileii*	NMI5622_11	NODE_44<br>NODE_33	38379<br>59857	Homo sapience	Tn*512*-like<br>Tn*5563a*
JAEHTA010000047.1<br>JAEHTA010000050.1	*P. monteileii*	PSB00047	-c0047 -<br>c0050	51891<br>44578	Homo sapience	ΔTn*512*<br>Tn*5563a*
JALLLY010000012.1<br>JALLLY010000110.1	*P. juntendi*	CCBH26530 12	-<br>-	85016<br>13939	Homo sapience	Tn*512*-like<br>Tn*5563a*
JAKGAU010000017.1<br>JAKGAU010000052.1	*P. juntendi*	AR 06	NODE_17<br>NODE_52	92387<br>33691	Water	*ant(2'')-Ic, aac(6')-I1,* ΔTn*501*, VIM- 2*
JALLLV010000025.1<br>JALLLV010000069.1	*P. juntendi*	CCBH28261 25<br>CCBH28261 69	-<br>-	84744<br>18370	Homo sapience	ΔTn*501**
BQIS01000035.1<br>BQIS01000046.1	*P. juntendi*	BML-PP051	seq 035<br>seq 046	58033<br>41590	Homo sapience	ΔTn*501**<br>Tn*5563a*
JPFC01000002.1	*P. aeruginosa*	T36994	contig 2	147637	Homo sapience	Tn*5563a*<br>*chrAB;* czc
CAADPP010000006.1<br>CAADPP010000335.1<br>CAADPP010000307.1	*P. aeruginosa*	DR-PA	NODE_8<br>NODE_397<br>NODE_366	27897<br>43323<br>12221	nd	nd
NSZE01000024.1<br>NSZE01000027.1	*P. aeruginosa*	AUS502	IPC239_24.1<br>IPC239_271	75966<br>51418	river, Australia	Tn*5393*<br>ΔTn*501*-like
DAHOBZ010000041.1<br>DAHOBZ010000119.1	*P. aeruginosa*	ARLG-10079	denovo.042<br>denovo.127	61557<br>6577	Homo sapience; Urine	Tn*5046*
QFNP01000030.1<br>QFNP01000051.1	*P. protegens*	52_018_000_R2	scaffold_370<br>scaffold_2650	74053<br>18400	hospital surfaces and	-

					sink samples	
AXUP01000462.1	*P. taiwanensis*	SJ9	contig 463	178072	industrial wastewater	Tn*5046* ,Cu-r, czc, Tn*5563a*
WIVU01000058.1<br>WIVU01000040.1	*P. helleri*	FSL R10-1637	NODE_58<br>NODE_40	39966<br>49747	pasteurized milk	-
JAEHTC010000076.1b<br>JAEHTC010000031.1	*P. koreensis*	PSB00045	c0076<br>c0031	30856<br>64709	Homo sapience, sputum	Tn*5563a*<br>ΔTn*501*
POGD01000006.1	*Pseudomonas sp*	MPR-R5B	NODE_6	117112	Groundwater	two mer-operons
JANFEZ010000008.1	*Pseudomonas sp*	Eb3	NODE_8	110480	Mangrove	ΔTn*501*
BCBA01000063.1	*Pseudomonas sp*	NBRC 111124	PPU10_CON00065	100622	culture collection	partial mer-operon
JAJSRO010000019.1	*Pseudomonas sp*	NMI542	15 NODE	129844	Homo sapience, rectal swab	Tn*512*-like 2 copies *VIM-2*
RHQS01000004.1	*Pseudomonas sp*	P99	contig 4	108977	nursing call button	Tn*5563a*
AEWE02000020.1	*Pseudomonas sp*	TJI-51	contig 20	104101	nursing call button	ΔTn*501**
QJRK01000012.1<br>++QJRK01000062.1	*Pseudomonas sp*	MB-090624	NODE_12<br>NODE_62	119699<br>34971	Lake Michigan	ΔTn*501**, Tn*5563a, chrAB*
JACNDO010000013.1	*Pseudomonas sp*	FW301-21D1A	contig 13	116955	Groundwater	Tn*501*-like
