# Supplementary figures and images for: Conjugative Plasmid pPPUT-Tik1-1 from a Permafrost Pseudomonas putida Strain and Its Present-Day Counterparts Inhabiting Environments and Clinics
Source: Int J Mol Sci. 2023 Aug 31;24(17):13518. doi: 10.3390/ijms241713518 (PMC10488154; doi:10.3390/ijms241713518)

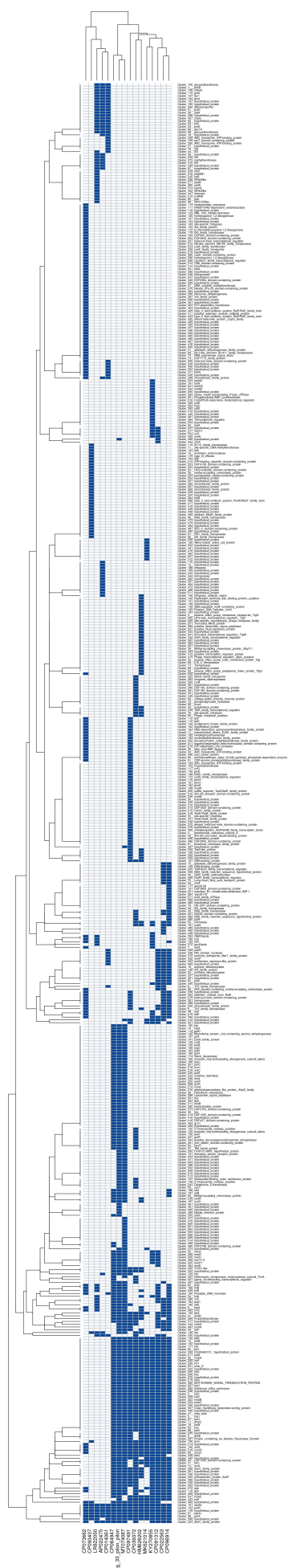

Supplement: Supplementary file 1 [file ijms-24-13518-s001.zip › Figure S1.pdf]

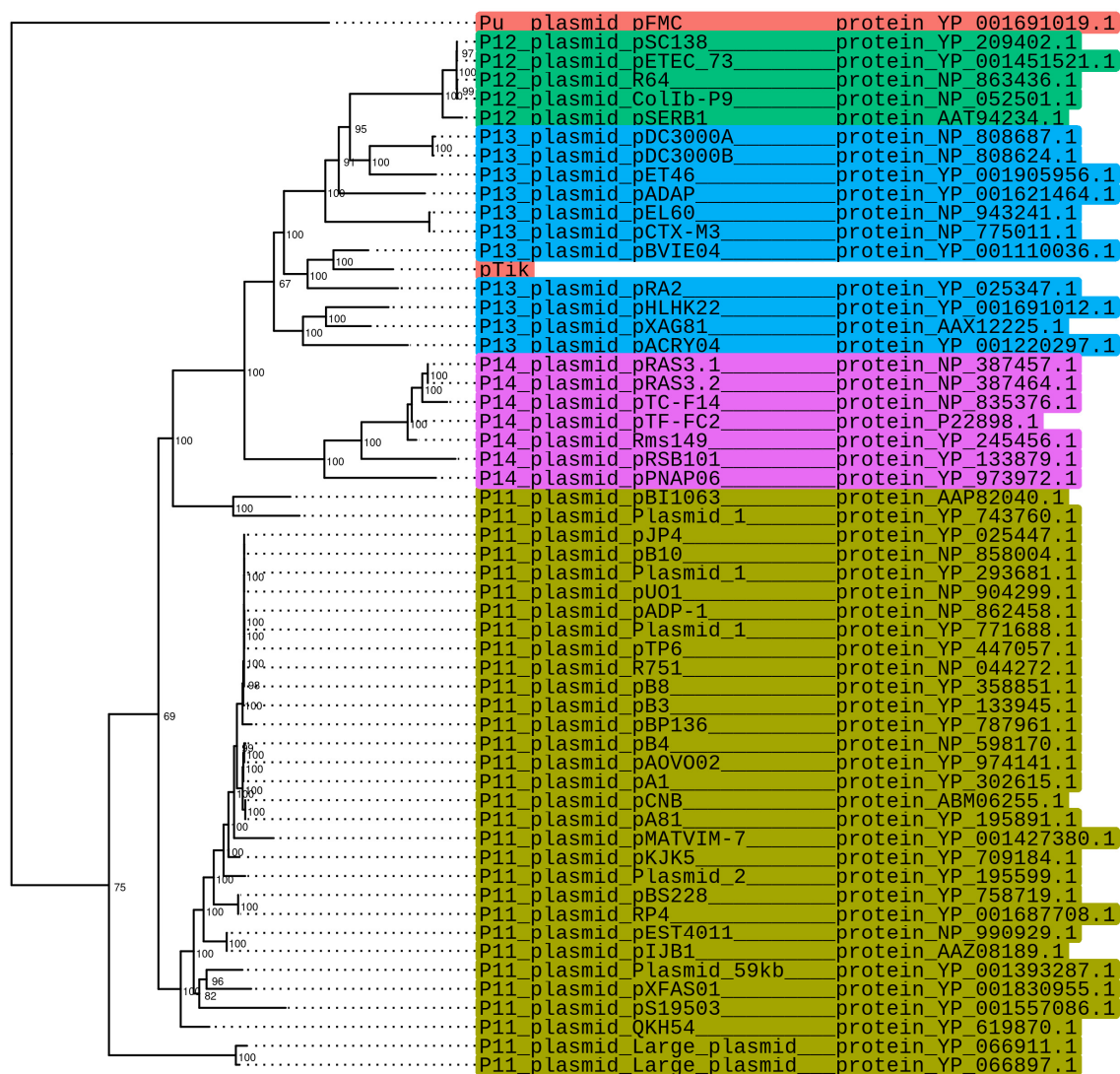

Figure S2. Phylogenetic tree of the *mob* genes from clades P11, P12, P13, P14.

Supplement: Supplementary file 1 [file ijms-24-13518-s001.zip › Figure S2.pdf]
